# Draft Genome Sequence of Guar (Cyamopsis tetragonoloba L.) Microsymbiont *Rhizobium* sp. Strain RCAM05973

**DOI:** 10.1128/mra.00071-23

**Published:** 2023-05-04

**Authors:** Polina Guro, Pavel Ulianich, Andrey Belimov, Anna Sazanova, Irina Kuznetsova, Margarita Vishnyakova, Vera Safronova

**Affiliations:** a All-Russia Research Institute for Agricultural Microbiology (ARRIAM), St. Petersburg, Russian Federation; b Federal Research Center, Vavilov All-Russian Institute of Plant Genetic Resources, St. Petersburg, Russian Federation; University of Arizona

## Abstract

Here, we present the draft genome sequence of *Rhizobium* sp. strain RCAM05973 which was isolated from a Cyamopsis tetragonoloba (guar) root nodule. The genome contains 6,937,221 bp in 2 contigs and has a GC content of 60%.

## ANNOUNCEMENT

Guar is an important commercial legume grown mainly in the arid zones of India. It is also cultivated in areas with a similar semiarid climate, such as the United States, Sudan, Kenya, Pakistan, Australia, and southern regions of Russia (1). As a legume plant, guar can form a symbiosis with rhizobia, which fix atmospheric nitrogen within the root nodules and thus increase plant productivity and soil fertility (2, 3).

The strain RCAM05973 was isolated from the nodule of a guar plant cultivated in a pot with a soil sample collected in the Republic of Buryatia (Russian Federation). Seeds of guar were previously surface sterilized by treatment with 98% H_2_SO_4_ for 30 min. Plants were cultivated for 50 days in the growth chamber with 50% relative humidity and a two-level illumination/temperatures mode of night (dark, 18°C, and 8 h) and day (400 μmol m^−2^ s^−1^, 24°C, and 16 h). Then individual nodules were separated from the roots, sterilized for 1 min in 70% ethanol, rinsed, homogenized in sterile tap water, and plated onto Yeast Extract Mannitol Agar (YMA) medium (4). Colonies of the strain RCAM05973 appeared after 3 days of incubation at 28°C. The strain was purified by repeated cloning. For DNA extraction, one single colony was selected, which was incubated in the R2A broth for 48 h at 28°C. The genomic DNA without size selection was extracted using a DNeasy blood and tissue kit (Qiagen, Germany) according to manufacturer recommendations. Library preparation was performed using the 1D native barcoding genomic DNA protocol with ligation sequencing kit SQK-LSK109 and the native barcoding expansion kit (EXP-NBD104/114; Oxford Nanopore Technologies [ONT], UK). The long-read whole genome was sequenced using a MinION sequencer with flow cell R.9.4.1 (ONT). The reads were base called and demultiplexed using the Guppy basecaller (v.5.0.11) (5). For this genome, 34,071 raw reads and 514.1 Mbp were generated with a mean length 15,089 bp (read length *N*_50_, 25,137 bp) and a median Q score of 11.7. The quality control of raw reads was performed with Nanostat (v.1.6.0) (6). Genome assembly was performed using Flye (v.2.9), and the resulting assembly was polished twice (7). It generated 2 contigs with a genome length 3,998,924 bp and 2,938,176 bp, with a total size of 6.9 Mbp and 38-fold mean coverage. The quality of the assembly was evaluated with QUAST (v.5.0.2), the GC content was 60%, and the *N*_50_ value was 3,998,924 bp (8). The assembly was then circularized using Circlator (v.1.5.5) with the following parameters recommended for ONT assembling: “–merge_min_id 85 –merge_breaklen 1000” (9). Default parameters were used for all software unless otherwise noted. The RAST server (v.2.0; access date, 8 November 2022) was used for annotation of the RCAM05973 genome (10). The draft genome contains 7,392 coding sequences and 61 RNA genes. The JSpeciesWS service was used to determine taxonomic relatedness based on average nucleotide identity (ANI) (11). The closest species was Rhizobium lusitanum type strain P1-7 (GCA_900094565.1) with values of 92.44% ANI based on BLAST+ (ANIb) identity and 93.72% ANI based on MUMmer (ANIm) identity. Values of less than 95% suggest that the species affiliation cannot be determined.

### Data availability.

All data are available at GenBank under BioProject accession number PRJNA899814. The BioSample accession number is SAMN31669504. The raw MinION data can be found under number SRR23096115. Assembly accession numbers are JAPHQM010000001 and JAPHQM010000002.

## References

[1] Kuznetsova IG, Sazanova AL, Safronova VI, Popova JP, Sokolova DV, Tikhomirova N, Osledkin Y, Karlov DS, Belimov АА, All-Russian Research Institute for Agricultural Microbiology, Federal Agency for Scientific Organizations. 2018. Isolation and identification of root nodule bacteria from guar Cyamopsis tetragonoloba (L.) Taub. Sel’skokhozyaistvennaya Biol 53:1285–1293. doi:10.15389/agrobiology.2018.6.1285eng.

[2] MacMillan J, Adams CB, Trostle C, Rajan N. 2021. Testing the efficacy of existing USDA Rhizobium germplasm collection accessions as inoculants for guar. Ind Crops Prod 161:113205. doi:10.1016/j.indcrop.2020.113205.

[3] Chaudhary SR, Sindhu SS. 2016. Growth stimulation of clusterbean (Cyamopsis tetragonoloba) by coinoculation with rhizosphere bacteria and Rhizobium. Legum ResJ 39:1003–1012. doi:10.18805/lr.v0iOF.8605.

[4] Safronova VI, Kuznetsova IG, Sazanova AL, Kimeklis AK, Belimov AA, Andronov EE, Pinaev AG, Chizhevskaya EP, Pukhaev AR, Popov KP, Willems A, Tikhonovich IA. 2015. Bosea vaviloviae sp. nov., a new species of slow-growing rhizobia isolated from nodules of the relict species Vavilovia formosa (Stev.) Fed. Antonie Van Leeuwenhoek 107:911–920. doi:10.1007/s10482-015-0383-9. 25603982

[5] Wick RR, Judd LM, Holt KE. 2019. Performance of neural network basecalling tools for Oxford Nanopore sequencing. Genome Biol 20:129. doi:10.1186/s13059-019-1727-y. 31234903 PMC6591954

[6] De Coster W, D'Hert S, Schultz DT, Cruts M, Van Broeckhoven C. 2018. NanoPack: visualizing and processing long-read sequencing data. Bioinformatics 34:2666–2669. doi:10.1093/bioinformatics/bty149. 29547981 PMC6061794

[7] Kolmogorov M, Yuan J, Lin Y, Pevzner PA. 2019. Assembly of long, error-prone reads using repeat graphs. Nat Biotechnol 37:540–546. doi:10.1038/s41587-019-0072-8. 30936562

[8] Gurevich A, Saveliev V, Vyahhi N, Tesler G. 2013. QUAST: quality assessment tool for genome assemblies. Bioinformatics 29:1072–1075. doi:10.1093/bioinformatics/btt086. 23422339 PMC3624806

[9] Hunt M, Silva ND, Otto TD, Parkhill J, Keane JA, Harris SR. 2015. Circlator: automated circularization of genome assemblies using long sequencing reads. Genome Biol 16:294. doi:10.1186/s13059-015-0849-0. 26714481 PMC4699355

[10] Aziz RK, Bartels D, Best AA, DeJongh M, Disz T, Edwards RA, Formsma K, Gerdes S, Glass EM, Kubal M, Meyer F, Olsen GJ, Olson R, Osterman AL, Overbeek RA, McNeil LK, Paarmann D, Paczian T, Parrello B, Pusch GD, Reich C, Stevens R, Vassieva O, Vonstein V, Wilke A, Zagnitko O. 2008. The RAST server: Rapid Annotations using Subsystems Technology. BMC Genomics 9:75. doi:10.1186/1471-2164-9-75. 18261238 PMC2265698

[11] Richter M, Rosselló-Móra R, Glöckner FO, Peplies J. 2015. JSpeciesWS: a Web server for prokaryotic species circumscription based on pairwise genome comparison. Bioinformatics 32:929–931. doi:10.1093/bioinformatics/btv681. 26576653 PMC5939971

